# Impact of a tropical forest blowdown on aboveground carbon balance

**DOI:** 10.1038/s41598-021-90576-x

**Published:** 2021-05-28

**Authors:** K. C. Cushman, John T. Burley, Benedikt Imbach, Sassan S. Saatchi, Carlos E. Silva, Orlando Vargas, Carlo Zgraggen, James R. Kellner

**Affiliations:** 1 Institute at Brown for Environment and Society, Brown University, Providence, RI 02912 USA; 2 Department of Ecology and Evolutionary Biology, Brown University, Providence, RI 02912 USA; 3 Aeroscout GmbH, Hochdorf, Switzerland; 4 NASA-Jet Propulsion Laboratory, California Institute of Technology, Pasadena, CA 91109 USA; 5 Department of Geography, University of Maryland, College Park, MD 20742 USA; 6 Organization for Tropical Studies, La Selva Biological Station, San Pedro, Costa Rica; 7 Present Address: Smithsonian Tropical Research Institute, 0843-03092 Balboa, Ancón Republic of Panama

**Keywords:** Forest ecology, Tropical ecology, Carbon cycle

## Abstract

Field measurements demonstrate a carbon sink in the Amazon and Congo basins, but the cause of this sink is uncertain. One possibility is that forest landscapes are experiencing transient recovery from previous disturbance. Attributing the carbon sink to transient recovery or other processes is challenging because we do not understand the sensitivity of conventional remote sensing methods to changes in aboveground carbon density (ACD) caused by disturbance events. Here we use ultra-high-density drone lidar to quantify the impact of a blowdown disturbance on ACD in a lowland rain forest in Costa Rica. We show that the blowdown decreased ACD by at least 17.6%, increased the number of canopy gaps, and altered the gap size-frequency distribution. Analyses of a canopy-height transition matrix indicate departure from steady-state conditions. This event will initiate a transient sink requiring an estimated 24–49 years to recover pre-disturbance ACD. Our results suggest that blowdowns of this magnitude and extent can remain undetected by conventional satellite optical imagery but are likely to alter ACD decades after they occur.

## Introduction

Long-term field measurements show that tropical forests in South America and Africa have accumulated carbon in recent decades, acting as a carbon sink that appears to be saturating in the Amazon and Congo basins^1–4^. Accumulation of aboveground carbon in forests is predicted in response to rising atmospheric CO_2_^5,6^. An alternative, but not mutually exclusive, explanation is that the intact-forest sink is associated with recovery from previous disturbance, including both anthropogenic and natural disturbances^7,8^. Here we characterize aboveground carbon dynamics following a blowdown event—one type of natural disturbance relevant for many Neotropical areas^9^.

The area impacted by natural forest disturbance is power-law distributed^10–12^—most events are small, but rare episodic disturbance events influence up to thousands of square kilometers. Recovery times associated with these episodic events could be substantial but the importance of forest recovery from natural disturbance for overall carbon dynamics is contested^7,13–16^. One cause of uncertainty is the challenge of measuring the carbon consequences of events that are difficult or impossible to predict. Small, frequent disturbances are well-characterized by forest plots, but sparse plot networks are unlikely to observe episodic events. Alternately, the largest episodic disturbances can be detected using optical satellite data. A single squall-line storm in the Amazon can affect thousands of km^2^, killing hundreds of thousands of trees through convective downdrafts called blowdowns^17,18^. There is a particular lack of information for episodic disturbances of intermediate severity—creating forest gaps that are larger than those typically observed in the field (< 0.1 ha) but smaller than those studied using historical satellite data that are coarse in temporal and spatial resolution (> 30 ha)^14^. How long forests remain in a state of recovery following these events, and how well these events are currently characterized by remote sensing studies, remains poorly understood.

**Figure 1:**
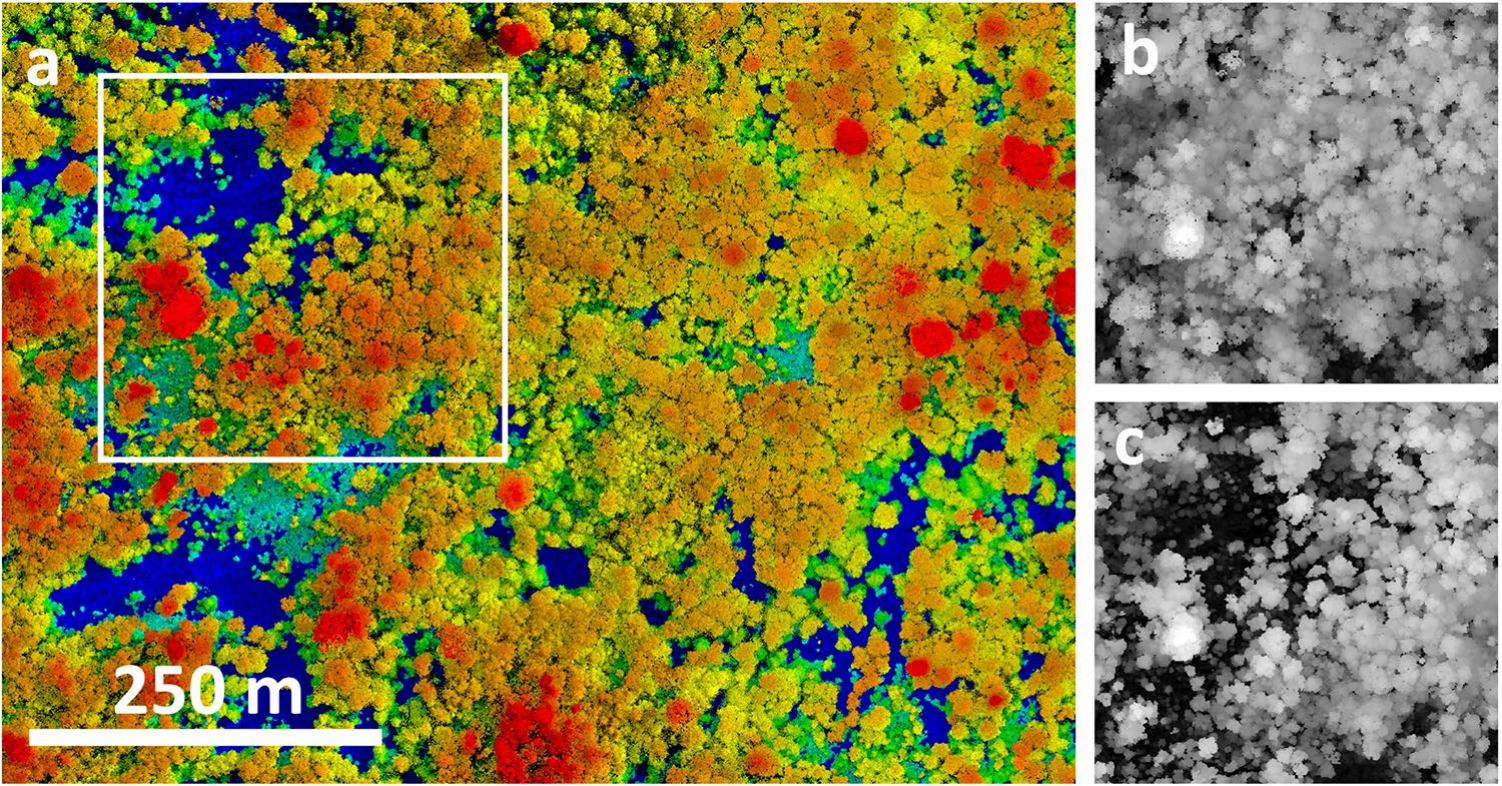
A tropical forest blowdown observed using high-resolution remote sensing. Images are from structure from motion photogrammetry (**a**) and lidar (**b**, **c**). Colors in (**a**) are surface elevation; brightness is scaled by RGB intensity. Inset shown in (**a**) is the area in (**b**) and (**c**). Colors in (**b**) and (**c**) are canopy height from airborne lidar in 2009 (**b**) and drone lidar in 2019 (**c**). Large height changes are apparent due to a blowdown event.

Here we take advantage of a large, infrequent disturbance in the Atlantic lowlands of Costa Rica to document aboveground carbon losses, canopy structural changes, and estimated recovery time. On May 19, 2018, a storm delivered 38 mm of rain in a 30-min period at La Selva Biological Station^19^. This blowdown event caused large-scale disturbance in forest structure, creating > 600 new gaps along trails alone, according to local field surveys (Supplementary Fig. 1)^19^. We collected ultra-high-density drone lidar data after the disturbance to quantify canopy structure and aboveground carbon density (ACD) over 103.5 ha of forest (33 ha in old-growth forest; 70.5 ha in secondary forest 31–66 years old in 2019; Fig. 1; Supplementary Fig. 2). By comparing these measurements to previously collected airborne lidar and inventory plot data, we identified multiple lines of evidence that indicate the disturbance was unprecedented in the previous 20 years of annual field measurements^20^. These measurements are used to address three main questions: 1) what is the impact of the blowdown disturbance on ACD and forest structure? 2) what is the estimated recovery time of the forest to its pre-disturbance ACD? 3) to what extent can similar events be detected by conventional remote sensing observations for large scale monitoring applications?

## Results and discussion

### Impact of disturbance on ACD and forest structure

We found that this blowdown disturbance caused substantial losses to ACD and changes to forest structure. The blowdown decreased mean ACD by 17.6% in comparison to pre-blowdown conditions, and average ACD loss was similar for old-growth (18.3%) and secondary forest areas (17.2%) in our study area (Supplementary Table 1). Carbon losses were heterogeneous throughout the forested landscape—some locations decreased in ACD by up to 63.1% while locations that were not impacted by the blowdown increased by up to 19.8% (Supplementary Fig. 1). We explored two potential causes of this spatial heterogeneity. First, we found no evidence that ACD loss was greater along trails (Supplementary Fig. 3), which is inconsistent with expectations that tree damage and mortality are greatest along forest edges^21^. This discrepancy is possible because local trails are likely not wide enough to increase wind exposure. Second, we found a weak correlation between ACD prior to the blowdown and ACD loss (Supplementary Fig. 4), which is consistent with previous research that found larger trees are more susceptible to damage in blowdowns^22^.

**Figure 2:**
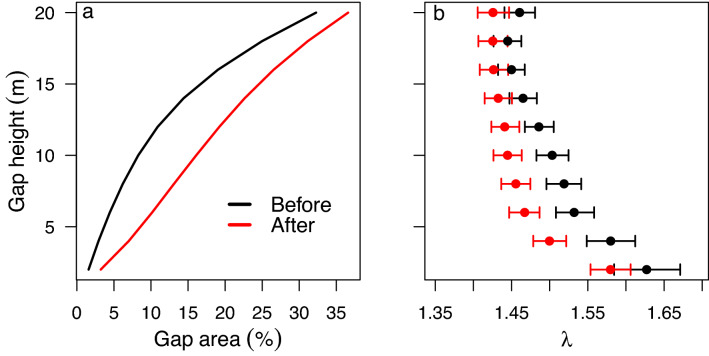
Total gap area (**a**) and the gap size-frequency distribution scaling exponent, $\uplambda$, (**b**) before and after the tropical forest moderate blowdown. Gap statistics were calculated using maximum height thresholds from 2 to 20 m in height in 2 m intervals. Gap area (**a**) is shown as a percentage of the total 103.5 ha study area. Error bars (**b**) denote the 95% Bayesian credible intervals for $\uplambda$.

The blowdown also significantly increased the size and frequency of canopy gaps, more than doubling the total forested area in gaps extending to ≤ 8 m height (Fig. 2a). The proportional increase in gap area was greater in secondary forests (arithmetic mean 82% increase) than in old-growth forest (mean 74% increase). The canopy gap size-frequency distribution was well described by a power-law probability distribution both before and after the blowdown, where most canopy gaps were small in size (Supplementary Fig. 5). The power-law scaling exponent ($\uplambda$) of the gap size-frequency distribution was consistently smaller after the blowdown for all gap height thresholds between 2 and 20 m, indicating an increased relative prevalence of larger gaps after the blowdown event. However, the decrease in $\uplambda$ was only significantly different for mid-canopy gaps extending to between 4 and 12 m height (Fig. 2b). Surprisingly, the size-frequency distribution of classically defined canopy gaps ≤ 2 m in height did not change significantly in response to the blowdown event^23^. Although classically defined gaps are useful because they can be easily quantified in ground surveys, our findings underscore the importance of high-resolution three-dimensional information to characterize the impacts of disturbance events in forest canopies (Fig. 1). Gap size-frequency results were qualitatively similar when old-growth and secondary forests were analyzed separately (Supplementary Fig. 6).

**Figure 3:**
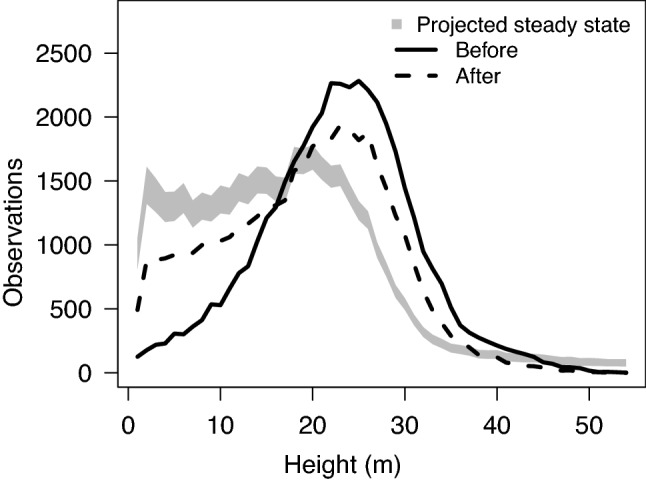
Distributions of canopy height (lines) before and after a moderate blowdown in the Atlantic lowlands of Costa Rica. The shaded region is the posterior of the projected distribution of canopy height based on the dominant right-hand eigenvector of a height transition matrix; the range of values at each height is the 95% Bayesian credible interval for the projected distribution.

Previous analyses demonstrated that canopy height and ACD changes in the old-growth forest landscape were consistent with steady state expectations^24,25^. We projected the equilibrium canopy height distribution using observed canopy height changes before and after the blowdown. The projected equilibrium is the expected distribution of canopy height if the canopy height transition probability matrix associated with the blowdown event persists indefinitely. By comparing the projected equilibrium distribution to the observed distribution prior to the blowdown event, we can test the hypothesis that the distribution of canopy height change associated with the blowdown event is a departure from steady-state conditions previously reported. Our projection of the equilibrium distribution of canopy height in response to the blowdown event was markedly different from the observed canopy height distribution and previous steady-state expectations (Fig. 3). The mean of the projected equilibrium canopy height was 15.9 m (95% confidence interval 15.7–16.1 m), substantially lower than the pre-blowdown mean height of 22.2 m (Supplementary Table 1), and lower than the steady-state canopy height of 19.6 m reported previously^24^. Projected equilibrium heights were qualitatively similar when old-growth and secondary forests in our study area were analyzed separately (Supplementary Fig. 7).

Our interpretation assumes that changes in the distribution of canopy height and ACD between 2009 and 2019 were driven by the 2018 blowdown event. Interval length can bias estimates of carbon stock change^26^. Here we likely underestimate ACD losses and structural changes attributable to the blowdown for two reasons. First, we expect that the landscape accumulated aboveground carbon between 2009 and the 2018 blowdown event. We expect this is true because our study area contains secondary forests, and because field measurements indicate that old-growth forests in this area steadily increased in ACD between 1997 and 2017^20^. We are also likely to underestimate ACD losses from the blowdown because post-blowdown lidar data were collected a year after the disturbance, and initial recovery can be rapid^11,21,27^. Consequently, we consider our conclusion regarding carbon loss and increased gap area to be conservative.

### Estimated duration of ACD recovery following disturbance

We used existing information about forest dynamics at this site to quantify the duration of the carbon sink (from forest regrowth) expected from this blowdown disturbance. We estimated recovery time by combining data from long-term field measurements at this site describing two processes: transient, rapid recovery following localized carbon losses (treefalls, Supplementary Fig. 8) and the long-term growth rate of ACD^20,28^. We estimate that carbon losses associated with this event will require 24–35 years to recover pre-disturbance mean ACD. Field measurements indicate an absolute increase in ACD of 0.7 Mg C ha^−1^ yr^−1^ between 2009 and 2016. Applying this increase to our estimate of pre-blowdown ACD increases the magnitude of carbon loss to 24% and increases the estimated recovery time to 38–49 years. Notably, these timeframes are longer than most field records of carbon accumulation in tropical forests. Two recent summaries of carbon balance in Amazonian and African tropical forests reported a mean monitoring period of 11–12 years^1,2^.

Our projected ACD recovery times assume that old-growth and secondary forests will regrow at the same rate. Previous studies of secondary forests in this landscape found that ACD is similar in old-growth forests and secondary forests older than 21 years, with rapid ACD recovery likely facilitated by the relatively fertile soils and proximity to intact forest^29,30^. Tree species richness and composition also stabilized within this time^29,31^. Our study area included only secondary forests older than 21 years at the beginning of our study (Supplementary Fig. 2); secondary forest ACD was within 93% of old-growth forest ACD at the beginning of our study (Supplementary Table 1). It is possible that secondary forest recovery rates will be slightly faster due to remaining compositional differences, such as lower liana abundance^32^.

### Potential for detection with conventional remote sensing methods

Although this disturbance reduced ACD by at least 17.6%, the blowdown was considerably smaller in size than larger reported blowdowns in the Amazon—the largest gaps we report are ~ 1 ha in size (Supplementary Fig. 5) while other studies report single gaps over 10 times as large^17^. We asked whether conventional optical satellite methods used to characterize larger blowdowns are also useful for detecting more moderate blowdowns. To determine whether the event was detectible using conventional optical satellite data, we identified cloud-free Landsat observations before and after the blowdown and computed the change in non-photosynthetic vegetation (ΔNPV) in each pixel. ΔNPV is used to detect blowdowns because tree mortality increases the fraction of woody vegetation exposed to the sensor^9,11,21,22^. As expected, ΔNPV values were significantly positive—*i.e*. the fraction of NPV increased after the blowdown (*t* = 16.2, *DF* = 206, *P* < 0.001). However, the mean and maximum ΔNPV in our data (0.02 and 0.09, respectively) were smaller than ΔNPV values reported in other studies—in fact, these values are approximately one order of magnitude smaller than those reported for large blowdowns^9^, and the mean value is within the 95% confidence interval for no mortality in one previous study^21^. Further, although we observed a statistically significant relationship between ΔNPV and ACD loss, the explanatory power of this relationship was very low (*r*^*2*^ = 0.03) (Supplementary Fig. 9). We attribute the difficulty of detecting this blowdown using Landsat to a combination of factors. Relatively low temporal frequency associated with high cloud cover resulted in cloud-free observations 6 and 7 months before and after the blowdown event, respectively. Tropical forests regrow quickly after blowdown events—even severe blowdowns are not detectable after < 2 years^11^.

### Prospective importance for tropical forest ACD dynamics

Results from this study are consistent with the idea that increasing ACD in tropical forest plots could, in part, reflect recovery from natural disturbance events that predate the onset of forest monitoring and remain cryptic to historical satellite records. This explanation does not preclude other potential causes of increasing ACD, such as carbon fertilization from rising atmospheric CO_2_ and widespread prior anthropogenic disturbance. The magnitude of ACD loss and estimated ACD recovery reported here are specific to this event and not directly generalizable to other areas because blowdown intensity and recovery from disturbance are both highly variable. Instead, this case study is valuable for highlighting that moderate episodic events can cause ACD losses of at least 17.6% while remaining undetectable using conventional measurement methods.

We anticipate that advances in satellite remote sensing—like Planet’s constellation of cube satellites and NASA’s Global Ecosystems Dynamics Investigation (GEDI) spaceborne lidar—will provide new opportunities to thoroughly characterize how other moderate blowdowns contribute to carbon cycling in global tropical forests^33–35^. For instance, mortality from the blowdown described here was clearly apparent in high-resolution (3 m) Planet data collected 8 days before and seven days after the blowdown event (Supplementary Fig. 1). The methodological framework presented here, in which a targeted airborne remote sensing campaign produced detailed measurements of ACD loss and structural changes, will be valuable for developing and benchmarking spaceborne measurements. Airborne lidar are increasingly available—such as > 100,000 ha of available tropical forest lidar data from the Sustainable Landscapes Brazil Project^36,37^, including multi-temporal lidar for some areas^38^—and will provide essential baseline data for future efforts. Our results suggest that incorporating widespread high-resolution monitoring of forest disturbance could increase the estimated aboveground carbon loss attributed to infrequent disturbance events in comparison to previous analyses limited to field records or historical satellite data^15^.

## Methods

### Study site

This study was conducted at La Selva Biological Station, located in the lowland Atlantic forest of Costa Rica (10°26′ N, 83°59′ W). The mean annual temperature is 26 °C; mean annual precipitation is 4 m and all months have mean precipitation > 100 mm^39^. La Selva has undulating topography, with elevation varying between 10 and 140 m above sea level. La Selva Biological Station includes multiple land uses; our analysis includes 103.5 hectares of forest, comprising 33.0 ha of old-growth forest and 70.5 ha of forests with past human disturbance (secondary forests, abandoned agroforestry, abandoned plantation, selectively-logged forests); here, we refer to all areas with past human disturbance as “secondary forests”. This study area does not include the full extent of old-growth or secondary forests at La Selva—we focused our drone data collection on this area because it contained the most severe apparent disturbance from the blowdown. Forests with past human disturbance have been naturally regenerating for a range of time (since 1955–1988); we excluded secondary forests with regeneration starting after 1988.

### Lidar data

We use two airborne lidar datasets to quantify dynamics in canopy structure and ACD. Data were collected in 2009 and 2019 (Supplementary Table 2). Data from 2009 were collected by a fixed-wing aircraft over the entire reserve; data from 2019 were collected using the Brown Platform for Autonomous Remote Sensing^40^. We focused on an area 1.4 km^2^ in size that includes the region of most severe damage from the blowdown (Supplementary Fig. 1). Both lidar sensors were discrete-return systems. To minimize variation in lidar height estimates from variable laser beam divergence and detector characteristics, we only used data from first returns for all analyses. For the 2019 drone-based lidar with higher native point density and a wider scan angle range^40^, we limited our analysis to lidar returns with scan angle ± 15 degrees and randomly subsampled data to a homogenous resolution of 10 pts m^−2^. Previous research demonstrates that lidar data collected above densities of 1 pts m^−2^ have similar predictive power for determining many forest properties (including tree height, tree density, and basal area)^41^; both lidar datasets in this study are above this density threshold. All lidar data were projected using EPSG 32,616.

For all lidar data, we calculated height above ground using a digital terrain model (DTM) created from lidar data collected in 2006 and validated using 4184 independent measurements within the old-growth forest (intercept =  − 0.406, slope = 0.999, r^2^ = 0.994, RMSE = 1.85 m; Supplementary Table 2)^42^. We verified that the horizontal geolocation accuracy with < 1 m between lidar datasets by comparing lidar returns from building roofs present in all datasets; we also used data from roof lines to adjust for a 0.7 m positive bias in the 2009 measurements relative to the 2019 lidar measurements (Supplementary Table 2).

### AGBD and ACD

We estimated aboveground biomass density (AGBD) for each lidar dataset using a model parameterized with 18 0.5 ha field plots established for the CARBONO project, in which plots were placed using a random stratified sampling design to characterize edaphic variation within La Selva^43^. All trees, lianas, and palms with diameter ≥ 10 cm at 1.3 m height are measured annually in CARBONO plots; field data from 2009 were used to parametrize the lidar-derived AGBD model. For each field plot, aboveground biomass was estimated for each stem using an allometric model including a regional diameter-height relationship and wood density^44^. We used wood density values at the most specific level possible (species, genus, family, or site-level mean). AGBD estimates do not include non-woody plant material, herbaceous plants, hemi-epiphytes, epiphytes, or woody stems smaller than 10 cm diameter. Omitted pools likely comprise < 15% of total AGBD^45^—the proportion of omitted pools may be larger for secondary forests, but we do not expect this to be an important source of bias because our study area includes only older secondary forests. Data and detailed methods for plot measurements are publicly available^28^.

We used a model relating top-of-canopy height (TCH) to AGBD using a power relationship:

1 $$AGBD=a{TCH}^{b}$$

where *a* and *b* are parameters fit using non-linear maximum likelihood analysis^46^. Maximum likelihood analysis was performed using the quasi-Newton method in the ‘stats4’ package in R (version 4.0.3), and assuming normally distributed residuals^47^. TCH was calculated by first computing the mean height of first lidar returns in every pixel of a 5 m × 5 m grid, and then computing the mean height of all pixels that fell within the boundaries of a single plot. This model explained 74% of variation in AGBD among field plots, with 9.2% RMSE (Supplementary Fig. 10). We assume that the relationship between TCH and AGBD was the same before and after the blowdown event. The distributions of model residuals showed no heteroscedasticity (Supplementary Fig. 10). Previous research indicates that a single relationship fits old-growth and secondary forests at La Selva^48^. To convert AGBD predictions into ACD, we multiplied AGBD by 0.47.^49^.

We applied this model to the 2009 and 2019 lidar datasets, using a 0.5 ha raster resolution corresponding to the field plot size. We used a Monte Carlo method, sampling 1000 times from the observed parameter values describing residual variation in the TCH to AGBD model, to quantify uncertainty (95% confidence intervals) in estimates of ACD and ACD change.

### Gap size-frequency distribution

We quantified the canopy gap size-frequency distribution for each lidar dataset by creating a canopy height model (CHM) with 1.25 m pixels^10^. To ensure that every pixel had a height value (in the occasional case where a pixel had no lidar returns), we created the canopy height model by using a Delaunay triangulation of first returns, gridded to 1.25 m resolution^50^. We defined gaps according to Brokaw’s classic definition: any contiguous area ≤ 2 m in height^23^, and we also repeated the analysis for a range of height thresholds up to 20 m height (in 2 m increments). We included diagonal pixels in our calculation of contiguous area.

We characterized the gap size-frequency distribution using the Zeta distribution, which is a discrete probability distribution, defined for integers $k$ ≥ 1, giving the probability that a gap contains *k* pixels:

2 $$f\left(k\right)= \frac{{k}^{-\lambda }}{\zeta \left(\lambda \right)}$$

where $\zeta \left(\lambda \right)$ is the Riemann zeta function. The parameter $\lambda$ is a power-law exponent describing the size-frequency distribution of gaps—small values of $\lambda$ indicate increased frequency of large gaps. Previous research indicates that the power-law Zeta distribution is appropriate for comparing gap sizes in tropical forests with diverse disturbance regimes^10^.

We estimated $\lambda$ using the Metropolis–Hastings algorithm within a Markov chain Monte Carlo (MCMC) procedure. Our analysis produces a Bayesian estimate of $\lambda$. We used an uninformative prior (uniform distribution between 1.01 and 5); this conservative potential range for $\lambda$ was chosen based on results from Kellner and Asner (2009). We used a random normal proposal density, with a mean equal to the previous iteration of $\lambda$ and standard deviation equal to 0.1; proposals of $\lambda$ < 1 were replaced with a new random proposal. We used a chain of length 100,000, discarded the first 5000 observations the burn-in period, and thinned the chain by using every 25^th^ value. We used the remaining 3800 values to determine the posterior median and 95% Bayesian credible intervals for the power-law exponent, $\uplambda$. Results from our entire study area (old-growth and secondary forests combined) are reported in the main text; we also repeated this analysis for old-growth and secondary forest areas separately (Supplementary Fig. 6).

### Canopy height change

We quantified the distribution of canopy height change between 2009 and 2019 using the 5 m resolution CHM, where canopy height was estimated using the mean height of all first lidar returns in a pixel; at this resolution, there were no pixels with no lidar returns. A forest in steady-state is expected to have mean canopy height change of approximately zero. A forest recovering from past disturbance is expected to have a mean canopy height change greater than zero, and a forest that experiences large disturbance during the interval between measurements is expected to have a mean canopy height change less than zero^24^. We calculated the distribution of canopy height change between 2009 and 2019 by subtracting the initial height of a pixel from the final height of a pixel (Supplementary Fig. 11).

The steady-state canopy height distribution of a forest is the expected distribution of canopy height if observed transition probabilities do not change. To calculate the projected steady-state canopy height distributions for each time interval, we created a canopy height transition matrix, $A$, with 54 rows and 54 columns. Each row and column of $A$ corresponds to a single 1-m height class, and the maximum value (54 m) was selected from the maximum height of any pixel. An entry from row *i* and column *j* in $A$, *a*_*ij*_, represents the number of pixels that were in height class *j* at the beginning of the time interval and in height class *i* at the end of the time interval. The projected steady-state height distribution is then obtained using an eigenvector decomposition:

3 $$A\mathbf{x}={\varvec{\uplambda}}\mathbf{x}$$

where $\mathbf{x}$ is the right-hand eigenvector, and ${\varvec{\uplambda}}$ is the eigenvalue (not to be confused with the power-law exponent used to characterize the gap size frequency distribution). Here, $\mathbf{x}$ gives the distribution of canopy heights for which applying the canopy height transition matrix results in no overall change in the distribution of heights. We used a Bayesian procedure to quantify uncertainty in our projections of steady-state canopy heights. Specifically, we assume that height transition probabilities (*i.e.* the columns of $A$) follow a multinomial distribution. The multinomial distribution has a conjugate prior distribution, the Dirichlet distribution, from which the posterior distribution can be solved numerically^51^. We assumed an uninformative Dirichlet prior, which is equivalent to assuming that height transitions are uniformly distributed across prospective height classes. We sampled from the posterior distribution of each height class 10,000 times to determine the 95% Bayesian credible intervals of the projected steady-state canopy height distribution. Results from our entire study area (old-growth and secondary forests combined) are reported in the main text; we also repeated this analysis for old-growth and secondary forest areas separately (Supplementary Fig. 7).

### Blowdown detection from Landsat imagery

 To assess whether this blowdown event could be detected using Landsat imagery, we compared Landsat 8 images before and after the blowdown event. We manually chose Landsat images that were closest in time to the event without cloud cover over our area of interest. These images came from November 10, 2017 (190 days before the blowdown) and December 31, 2018 (226 days after the blowdown). We used the Landsat 8 Surface Reflectance Tier 1 data product, which is atmospherically corrected using United States Geological Survey Land Surface Reflectance Code. We then performed a Spectral Mixture Analysis (SMA) with endmembers for photosynthetic vegetation, non-photosynthetic vegetation (NPV), and shade; previous studies have shown that the change in proportion of NPV per pixel correlates with blowdown mortality and tree damage^9,17,21,22^. Because no pure pixels of NPV were apparent in our image, we used the tropical forest Landsat endmembers published by Schwartz et al. (2017). SMA was performed using ENVI’s linear spectral unmixing tool, using the constraint that endmembers must sum to one^52^. We compared the change in NPV to the change in ACD across our study landscape (Supplementary Fig. 9).

### Estimated recovery time from disturbance

We estimated recovery time required for the landscape to recover to its pre-blowdown ACD using the CARBONO project plot data^43^. Tropical forest AGBD recovery following disturbance is highly variable and depends on multiple factors including the severity of disturbance, climate, and soils^53,54^. Any approach to estimate recovery time will be highly uncertain due to unknowable factors such as future disturbance and climate, so we used two approaches that are likely to over- and under-estimate recovery time to approximate the range of likely recovery times.

First, we estimated recovery time using the long-term annual ACD gain in old-growth forests at our study site from 1997 to 2016. This long-term gain was previously reported in^20^ using the allometry of^55^. Here we report the long-term annual gain of 0.49 Mg C ha^−1^ yr^−1^ using the allometry of^44^. Because our analysis indicates a mean ACD loss of 17.4 Mg C ha^−1^ (Supplementary Table 1), the long-term ACD trend predicts 35 years to recover from the blowdown. We expect that this overestimates recovery time because ACD gain directly after the disturbance event should be greater than the long-term average.

Second, we estimate recovery time by incorporating fast initial ACD gain following large treefalls. We calculated the average ACD gain following the four observations in the CARBONO record where the annual ACD loss was > 11.5 Mg C ha^−1^. For 5 years following these disturbance events, annual ACD gain was larger in magnitude than the long-term trend (Supplementary Fig. 8). We estimated recovery time assuming these elevated post-disturbance recovery rates for the first 5 years, and then assuming the long-term gain for subsequent years, resulting in a predicted recovery time of 24 years. We expect that this underestimates recovery time because not all parts of the landscape experienced large decreases in ACD, but this method assumes that the entire landscape will have elevated ACD gain following the disturbance.

Finally, we repeated both approaches above, additionally estimating a higher value for pre-blowdown ACD in 2018 because plot based observations at La Selva indicate that ACD increased between 2009 and 2016^20^. We calculated that the average annual ACD gain between 2009 and 2016 was 0.74 Mg C ha^−1^ yr^−1^. To estimate ACD in 2018 immediately prior to the blowdown, we added this average rate to our 2009 ACD estimates for the period of 2009–2018. We subsequently re-estimated recovery time to the higher 2018 ACD estimate, resulting in recovery time between 38 and 48 years using the second and first approaches above, respectively.

## Supplementary Information

Supplementary Information.

## Data Availability

Canopy height data and code underlying this analysis are available at https://github.com/kccushman/LaSelvaBlowdown; the repository contents at the time of submission are also permanently archived through the Brown University Dataverse (hosted by the Harvard Dataverse) at https://doi.org/10.7910/DVN/VHW3XR.

## References

[1] Hubau W (2020). Asynchronous carbon sink saturation in African and Amazonian tropical forests. Nature.

[2] Brienen RJW, Phillips OL, Feldpausch TR (2015). Long-term decline of the Amazon carbon sink. Nature.

[3] Lewis SL (2009). Increasing carbon storage in intact African tropical forests. Nature.

[4] Pan Y (2011). A large and persistent carbon sink in the world’s forests. Science.

[5] Cernusak LA (2013). Tropical forest responses to increasing atmospheric CO2: current knowledge and opportunities for future research. Funct. Plant Biol..

[6] Hofhansl F (2016). Amazon forest ecosystem responses to elevated atmospheric CO2 and alterations in nutrient availability: Filling the gaps with model-experiment integration. Front. Earth Sci..

[7] Fisher JI, Hurtt GC, Thomas RQ, Chambers JQ (2008). Clustered disturbances lead to bias in large-scale estimates based on forest sample plots. Ecol. Lett..

[8] De Souza JG (2018). Pre-Columbian earth-builders settled along the entire southern rim of the Amazon. Nat. Commun..

[9] Negrón-Juárez RI (2018). Vulnerability of Amazon forests to storm-driven tree mortality. Environ. Res. Lett..

[10] Kellner JR, Asner GP (2009). Convergent structural responses of tropical forests to diverse disturbance regimes. Ecol. Lett..

[11] Nelson BW (1994). Forest disturbance by large blowdowns in the Brazilian Amazon. Ecology.

[12] Chambers JQ (2013). The steady-state mosaic of disturbance and succession across an old-growth central Amazon forest landscape. Proc. Natl. Acad. Sci. U.S.A..

[13] Lloyd J, Gloor EU, Lewis SL (2009). Are the dynamics of tropical forests dominated by large and rare disturbance events?. Ecol. Lett..

[14] Chambers JQ, Negrón-Juárez RI, Hurtt GC, Marra DM, Higuchi N (2009). Lack of intermediate-scale disturbance data prevents robust extrapolation of plot-level tree mortality rates for old-growth tropical forests. Ecol. Lett..

[15] Espírito-Santo FDB (2014). Size and frequency of natural forest disturbances and the Amazon forest carbon balance. Nat. Commun..

[16] Gloor M (2009). Does the disturbance hypothesis explain the biomass increase in basin-wide Amazon forest plot data?. Glob. Chang. Biol..

[17] Negrón-Juárez, R. I. *et al.* Widespread Amazon forest tree mortality from a single cross-basin squall line event. *Geophys. Res. Lett.* **37**, L16701 (2010).

[18] Garstang M, Massie HL, Halverson J, Greco S, Scala J (1994). Amazon coastal squall lines. Part I: structure and kinematics. Mon. Weather Rev..

[19] Rader AM (2020). Tree functional traits as predictors of microburst-associated treefalls in tropical wet forests. Biotropical.

[20] Clark DB (2019). Diversity, distribution and dynamics of large trees across an old-growth lowland tropical rain forest landscape. PLoS ONE.

[21] Schwartz NB (2017). Fragmentation increases wind disturbance impacts on forest structure and carbon stocks in a western Amazonian landscape. Ecol. Appl..

[22] Rifai SW (2016). Landscape-scale consequences of differential tree mortality from catastrophic wind disturbance in the Amazon. Ecol. Appl..

[23] Brokaw NVL (1982). The definition of treefall gap and Its effect on measures of forest dynamics. Biotropica.

[24] Kellner JR, Clark DB, Hubbell SP (2009). Pervasive canopy dynamics produce short-term stability in a tropical rain forest landscape. Ecol. Lett..

[25] Dubayah RO (2010). Estimation of tropical forest height and biomass dynamics using lidar remote sensing at La Selva, Costa Rica. J. Geophys. Res. Biogeosci..

[26] Muller-Landau HC, Detto M, Chisholm RA, Hubbell SP, Condit R, Coomes D, Burslem D, Simonson W (2014). Detecting and projecting changes in forest biomass from plot data. Forests and Global Change.

[27] Silvério DV (2019). Fire, fragmentation, and windstorms: a recipe for tropical forest degradation. J. Ecol..

[28] Clark, D. A. & Clark, D. B. The CARBONO Project: Long-term landscape-scale monitoring of tropical rain forest productivity and dynamics (2019). www.ots.ac.cr/carbonoproject. (Accessed: 8th October 2019).

[29] Letcher SG, Chazdon RL (2009). Rapid recovery of biomass, species richness, and species composition in a forest chronosequence in Northeastern Costa Rica. Biotropica.

[30] Drake JB (2002). Estimation of tropical forest structural characteristics, using large-footprint lidar. Remote Sens. Environ..

[31] Chazdon RL (2007). Rates of change in tree communities of secondary Neotropical forests following major disturbances. Philos. Trans. R. Soc. B Biol. Sci..

[32] Guariguata MR, Ostertag R (2001). Neotropical secondary forest succession: changes in structural and functional characteristics. For. Ecol. Manag..

[33] Dubayah, R. *et al.* The global ecosystem dynamics investigation: high-resolution laser ranging of the earth’s forests and topography. *Sci. Remote Sens.* **1**, 100002 (2020).

[34] Michael Y (2018). Economic assessment of fire damage to urban forest in the wildland-urban interface using planet satellites constellation images. Remote Sens..

[35] Drusch M (2012). Sentinel-2: ESA’s optical high-resolution mission for GMES operational services. Remote Sens. Environ..

[36] Dos-Santos MN, Keller MM, Morton DC (2019). ORNL DAAC.

[37] Longo M (2016). Aboveground biomass variability across intact and degraded forests in the Brazilian Amazon. Global Biogeochem. Cycles.

[38] Leitold V (2018). El Niño drought increased canopy turnover in Amazon forests. New Phytol..

[39] McDade LA, Bawa KS, Hespenheide HA, La Hartshorn GS (1994). Selva: Ecology and Natural History of a Neotropical Rain Forest. La Selva: Ecology and Natural History of a Neotropical Rain Forest.

[40] Kellner JR (2019). New opportunities for forest remote sensing through ultra-high-density drone lidar. Surv. Geophys..

[41] Jakubowski MK, Guo Q, Kelly M (2013). Tradeoffs between lidar pulse density and forest measurement accuracy. Remote Sens. Environ..

[42] Kellner JR, Clark DB, Hofton MA (2009). Canopy height and ground elevation in a mixed-land-use lowland neotropical rain forest landscape. Ecology.

[43] Clark DA, Clark DB, Oberbauer SF (2013). Field-quantified responses of tropical rainforest aboveground productivity to increasing CO2 and climatic stress, 1997–2009. J. Geophys. Res. Biogeosci..

[44] Chave J (2014). Improved allometric models to estimate the aboveground biomass of tropical trees. Glob. Change Biol..

[45] Muller-Landau HC (2020). Patterns and mechanisms of spatial variation in tropical forest productivity, woody residence time, and biomass. New Phytol..

[46] Asner GP, Mascaro J (2014). Mapping tropical forest carbon: Calibrating plot estimates to a simple LiDAR metric. Remote Sens. Environ..

[47] R Core Team. R: A Language and Environment for Statistical Computing. (2019).

[48] Drake JB (2003). Above-ground biomass estimation in closed canopy Neotropical forests using lidar remote sensing: factors affecting the generality of relationships. Glob. Ecol. Biogeogr..

[49] Martin AR, Thomas SC (2011). A reassessment of carbon content in tropical trees. PLoS ONE.

[50] Roussel, J.-R. & Auty, D. lidR: Airborne LiDAR data manipulation and visualization for forestry applications. *R Packag. version 2.1.2* (2019).

[51] Clark JS (2007). Models for Ecological Data.

[52] Exelis Visual Information Solutions. ENVI version 5.2.1. (2019).

[53] Chazdon RL (2003). Tropical forest recovery: Legacies of human impact and natural disturbances. Perspect. Plant Ecol. Evol. Syst..

[54] Poorter L (2016). Biomass resilience of Neotropical secondary forests. Nature.

[55] Brown S (1997). Estimating biomass and biomass change of tropical forests: a primer. FAO For. Pap..

