# Supplementary material for: Impact of a tropical forest blowdown on aboveground carbon balance
Source: Sci Rep. 2021 May 28;11:11279. doi: 10.1038/s41598-021-90576-x (PMC8163810; doi:10.1038/s41598-021-90576-x)
Supplement: Supplementary file 1 — Supplementary Information. [file 41598_2021_90576_MOESM1_ESM.docx]

**Supplementary information for:**

**Impact of a tropical forest blowdown on aboveground carbon balance**

K. C. Cushman^1,2,3*^, John T. Burley^1,2^, Benedikt Imbach^4^, Sassan S. Saatchi^5^, Carlos E. Silva^6^, Orlando Vargas^7^, Carlo Zgraggen^4^, and James R. Kellner^1,2^

^1^Institute at Brown for Environment and Society, Brown University, Providence, RI 02912

^2^Department of Ecology and Evolutionary Biology, Brown University, Providence RI, 02912

^3^Present address: Smithsonian Tropical Research Institute, Apartado 0843-03092, Balboa, Ancón, Republic of Panamá

^4^Aeroscout GmbH, Hochdorf, Switzerland

^5^NASA-Jet Propulsion Laboratory, California Institute of Technology, Pasadena, CA, 91109

^6^Department of Geography, University of Maryland, College Park MD, 20742

^7^Organization for Tropical Studies, La Selva Biological Station, San Pedro, Costa Rica

**Supplementary Table 1. Canopy height and ACD before and after a moderate blowdown in a lowland tropical forest.** The study area includes 33.0 ha of old-growth forest and 70.5 ha of secondary forest at La Selva Biological Station, Costa Rica. Brackets denote 95% confidence intervals from Monte Carlo simulation of the height-to-ACD model.

| **Year** | **Before blowdown** | | | **After blowdown** | | | |
| --- | --- | --- | --- | --- | --- | --- | --- |
| **Forest type** | All | Old growth | Secondary | All | Old growth | Secondary | |
| **Mean ACD** (Mg C ha^-1^) | 98.8 [97.5, 99.9] | 103.3 [101.1, 105.7] | 96.6 [95.0, 98.1] | 81.4 [80.1, 82.6] | 84.4 [82.4, 86.6] | | 80.0 [78.5, 81.5] |
| **Std. dev. ACD** (Mg C ha^-1^) | 15.1  [14.0, 16.2] | 15.2  [13.2, 17.2] | 14.6  [13.2, 16.0] | 17.8  [16.7 18.9] | 16.2  [14.1, 18.1] | 18.4  [16.9, 19.8] | |
| **Mean canopy height** (m) | 22.2 | 23.2 | 21.6 | 18.4 | 19.0 | 18.1 | |

**Supplementary Table 2. Characteristics of lidar data from La Selva Biological Station, Costa Rica, used in this study**. Lidar data from 2006 were used to create a digital terrain model, and lidar data from 2009 and 2019 were used to compare aboveground biomass density (AGBD) before and after a blowdown event.

| **Collection date** | **Sensor** | **Height adjustment (m)** | **Reference** |
| --- | --- | --- | --- |
| Mar., 2006 | Leica ALS50 | - 0.5 | (1) |
| Sep.-Oct., 2009 | Optech 3100 EA | + 0.7 | (2) |
| May, 2019 | Riegl VUX-1 | + 0.0 | (3) |

**
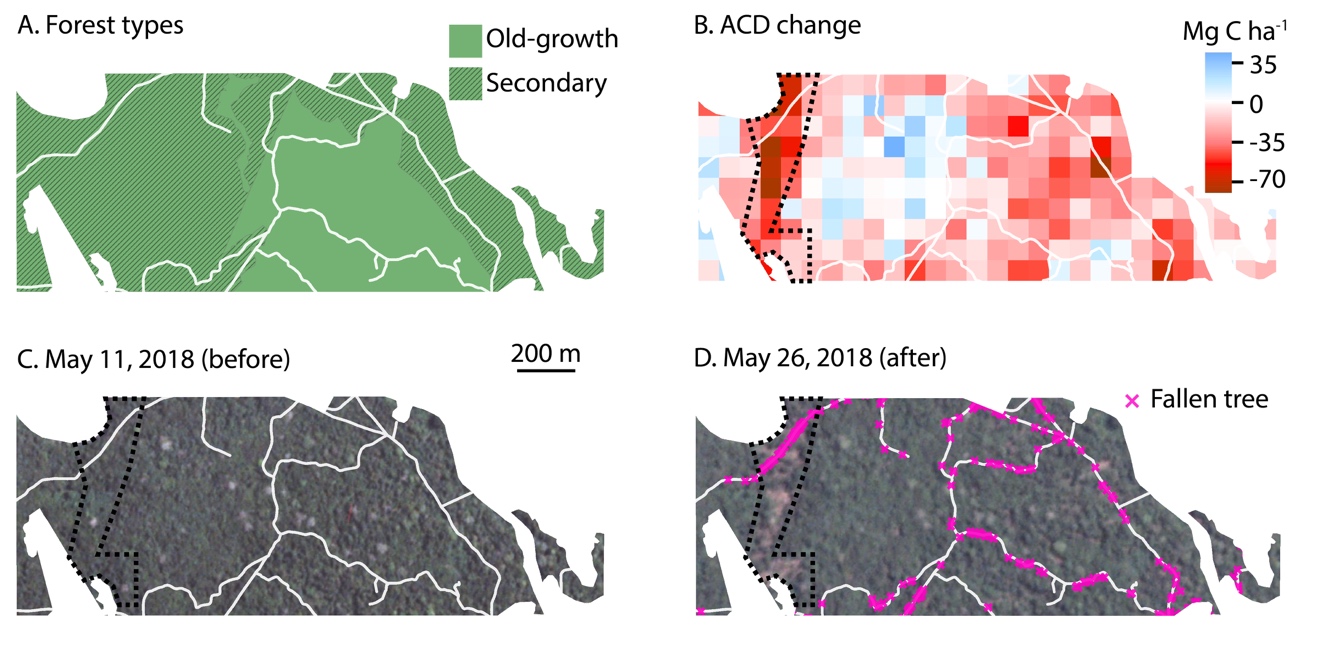
**

**Supplementary Figure 1**. Study area at La Selva Biological Station (A), and the magnitude of May 2018 blowdown as seen from lidar estimates of aboveground carbon density (ACD) loss (B) and Planet Labs imagery on May 11 (C, before blowdown) and May 26 (D, after blowdown). A ground survey of all trails (white lines) conducted immediately following the blowdown identified trees that fell across regularly maintained trails during the blowdown (D). Non-trail areas were not included in the post-disturbance ground survey. A region where blowdown damage is apparent in Planet imagery is denoted with a dashed line in B-D.

**Supplementary Figure 2**. Forest classification (A) of study area, and year of abandonment for secondary forest types (B).

**Supplementary Figure 3**. Comparison of ACD change in pixels with trails versus pixels without trails. Mean ACD change values for each category are denoted with vertical red lines. The distribution of ACD change values were not significantly different for pixels with and without trails, either with a t-test (*t* = -1.07, *DF* = 231.4, *P* = 0.29) or a Kolmogorov-Smirnov test (*D* = 0.169, *P* = 0.08).

**Supplementary Figure 4.** Relationship between ACD loss during the blowdown and pre-blowdown ACD. Results are shown for absolute (A) and proportional (B) ACD loss; both linear relationships are significantly negative (panel A: *DF* = 241, *R^2^* = 0.10, *P* < 0.001; panel B: *DF* = 241, *R^2^* = 0.02, *P* = 0.02).


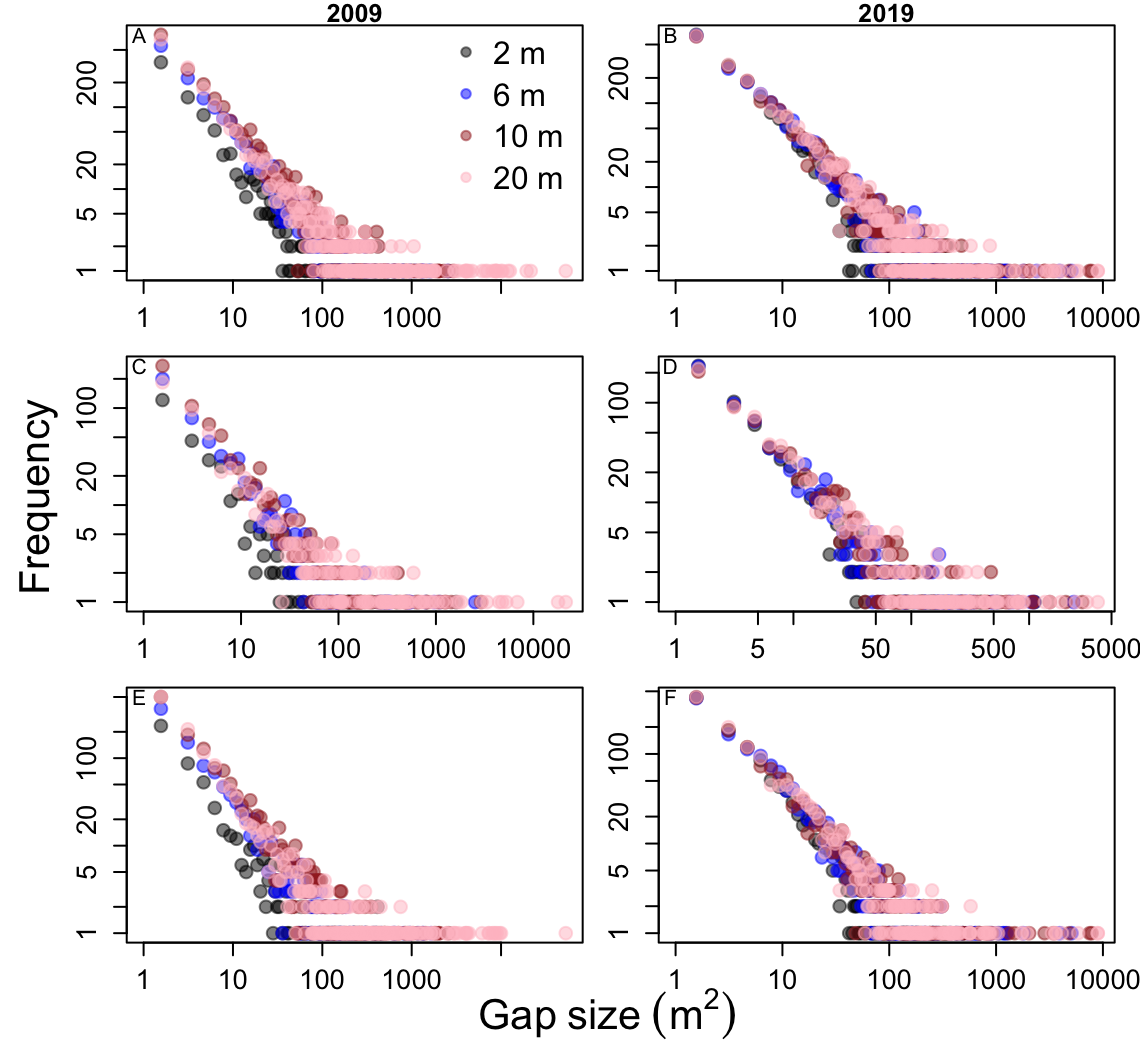


**Supplementary Figure 5**. Gap size-frequencies in La Selva Biological Station, quantified using airborne lidar before (2009) and after (2019) the May 19, 2018 blowdown at La Selva Biological Station, Costa Rica. Data are shown for 4 gap height thresholds (2, 6, 10, and 20 m; maximum vegetation height in gaps). Results are shown for all forest (A, B), and separately for only old-growth forest (C, D) or only secondary forest (E, F).


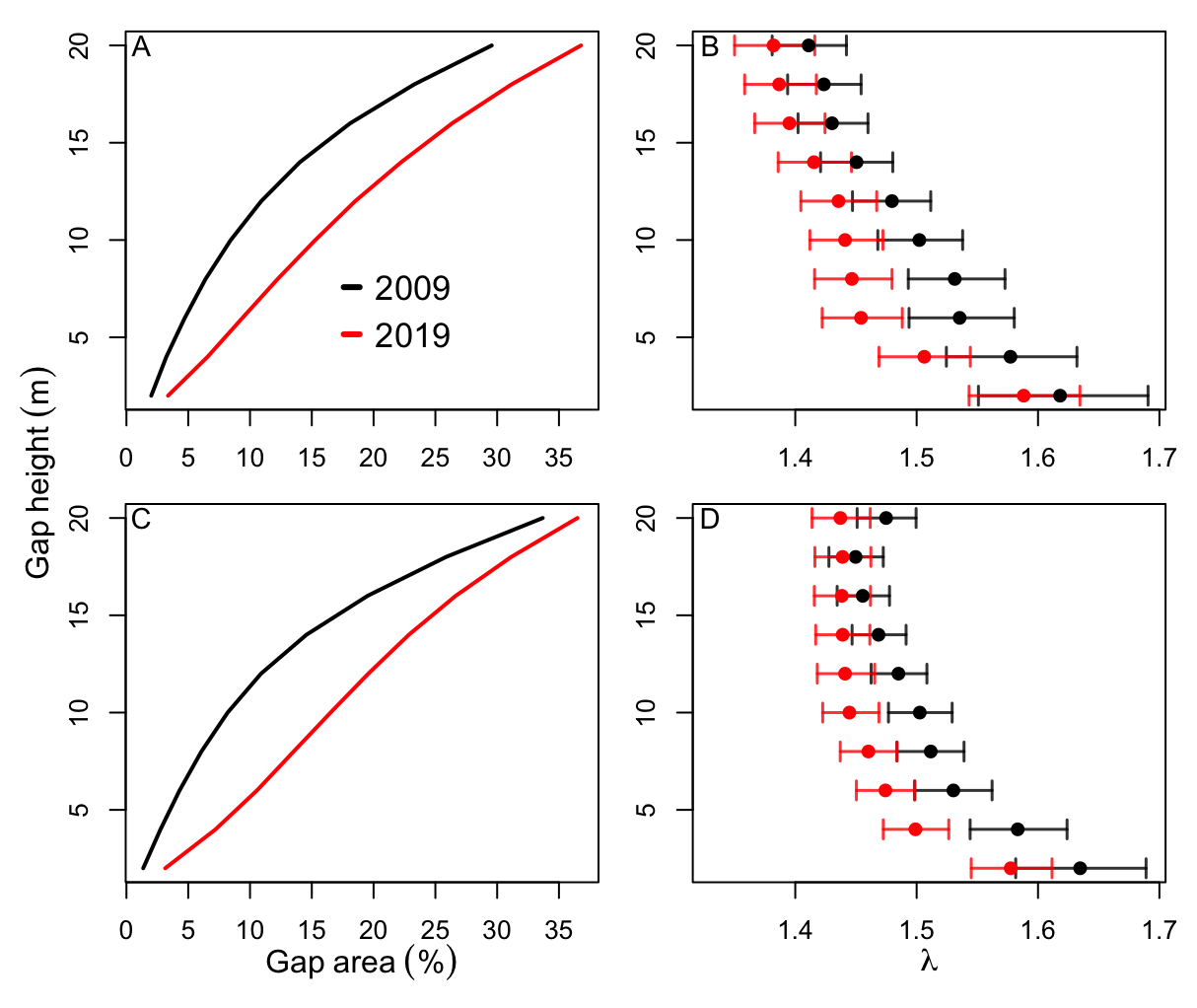


**Supplementary Figure 6**. Total gap area (A, C) and the gap size-frequency distribution scaling exponent, $\lambda$, (B, D) before and after the May 19, 2018 blowdown at La Selva Biological Station, Costa Rica. Results are shown separately for old-growth (A, B) and secondary forests (C, D). Gap statistics were calculated using a range of gap height thresholds (maximum vegetation height in gaps) from 2 to 20 m in height, in 2 m intervals. Gap area (A,C) is shown as a percentage of the total ha study area.


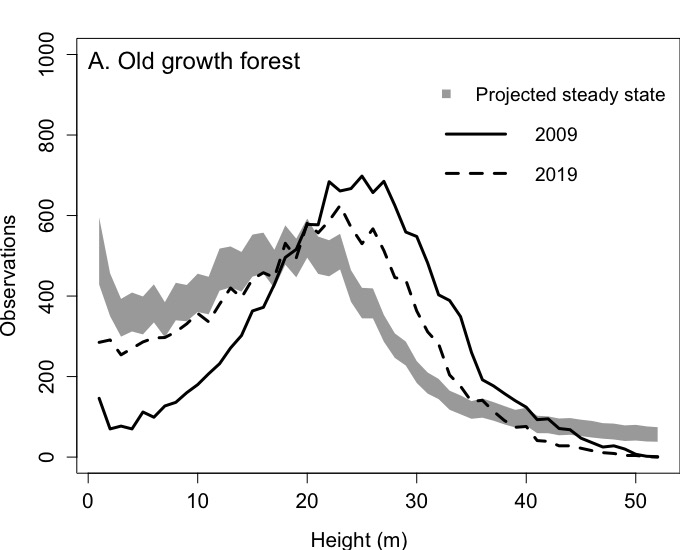

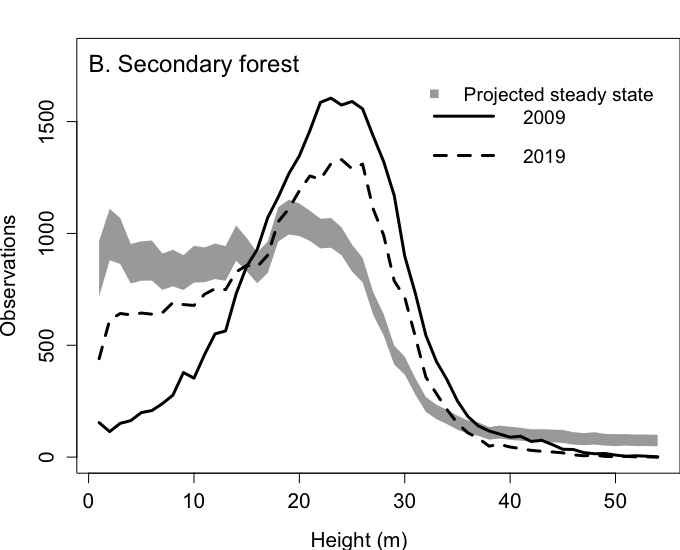


**Supplementary Figure 7**. Distributions of canopy heights (lines) measured by lidar before (2009) and after (2019) the May 19, 2018 blowdown at La Selva Biological Station, Costa Rica. Separate analyses are shown for old-growth (A) and secondary (B) forests. Projected steady state canopy height distributions, from canopy height dynamics between 2009 and 2019, are shown in a shaded line.


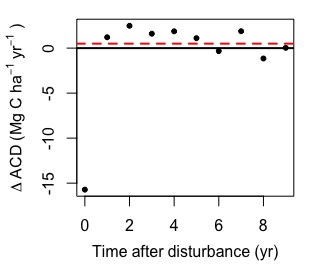


**Supplementary Figure 8.** Average AGBD recovery across following the four disturbance events that caused annual AGBD loss > 11.5 Mg ha^-1^ in the CARBONO record. The long-term annual average AGBD gain from 1997-2016 in the CARBONO record is denoted with a dashed red line. The five years following disturbance were used to estimate recovery time following the 2018 La Selva blowdown.

**
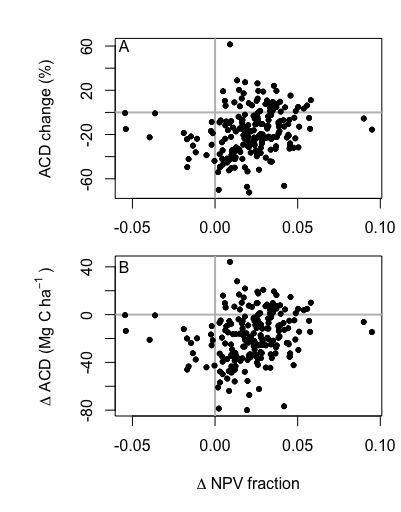
**

**Supplementary Figure 9.** Distribution of the change in proportion of non-photosynthetic vegetation (NPV) in Landsat data compared to the percentage of aboveground carbon density (ACD) change (A) and absolute biomass change (B) after the 2018 blowdown at La Selva Biological Station, Costa Rica. Landsat data come from the nearest cloud-free images before (November 2017) and after (December 2018) the blowdown event (May 2018), and ACD change is from 2009-2019. Landsat data (30 m resolution) were sampled to the resolution of AGBD data (0.5 ha) using a bilinear interpolation.

**
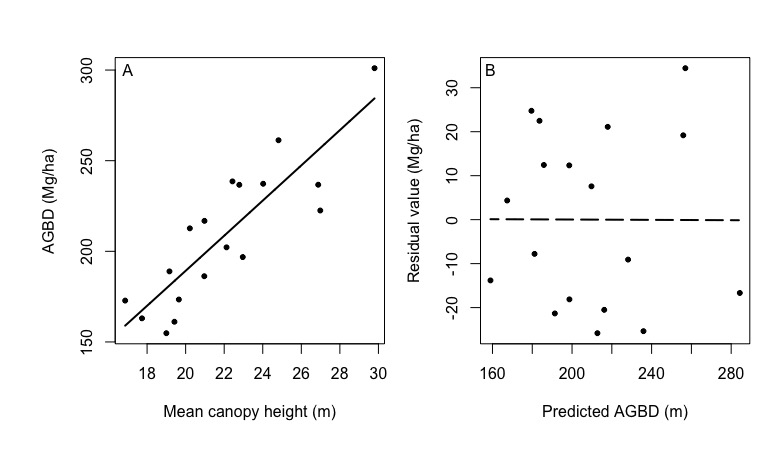
**

**Supplementary Figure 10.** Relationship between lidar top-of-canopy height (TCH) and AGBD (A), and the relationship between predicted AGBD and model residuals (B). TCH and AGBD were related using a power relationship ($AGBD=a{TCH}^{b}$), where *a* = 8.881 and *b* = 1.021, that explained 74% of the variation among plots with 9.2% RMSE. There was no significant linear relationship between predicted AGBD and residual values (*DF* = 16, *F* = 0.0002, *P* = 0.99).


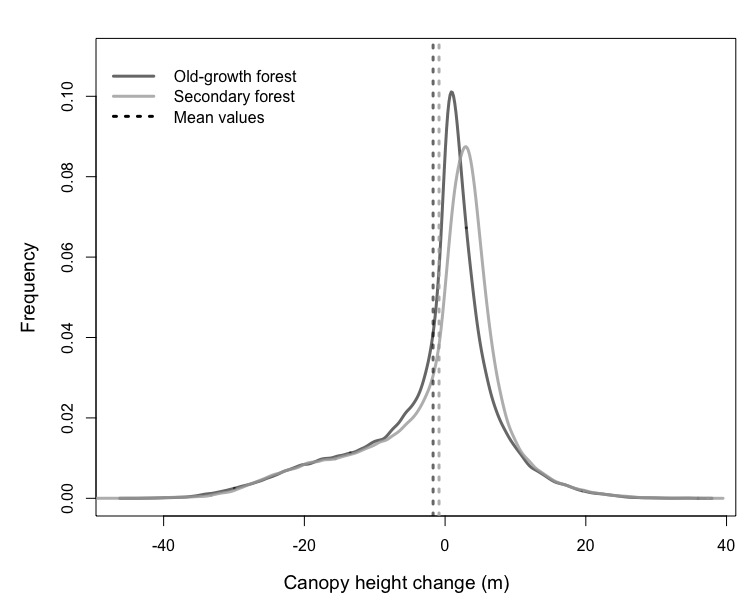


**Supplementary Figure 11.** Distribution of canopy height change across La Selva Biological Station, Costa Rica, between 2009 and 2019. Data from 2019 were collected approximately one year after the 2019 blowdown event. The vertical dashed lines denote the mean value for each interval. Old growth and secondary forests are separate for each year.
